# Evaluation of pulmonary dysfunctions and acid–base imbalances induced by *Chlamydia psittaci* in a bovine model of respiratory infection

**DOI:** 10.1186/2049-6958-9-10

**Published:** 2014-02-11

**Authors:** Carola Ostermann, Susanna Linde, Christiane Siegling-Vlitakis, Petra Reinhold

**Affiliations:** 1 Institute of Molecular Pathogenesis at ‘Friedrich-Loeffler-Institut’ (Federal Research Institute for Animal Health), Naumburger Str. 96a, 07743 Jena, Germany; 2 Freie Universität Berlin, Faculty of Veterinary Medicine, Oertzenweg 19b, 14163 Berlin, Germany

**Keywords:** Acid–base status, Animal model, *Chlamydia psittaci*, Pulmonary function

## Abstract

**Background:**

*Chlamydia psittaci* (*Cp*) is a respiratory pathogen capable of inducing acute pulmonary zoonotic disease (psittacosis) or persistent infection. To elucidate the pathogenesis of this infection, a translational large animal model was recently introduced by our group. This study aims at quantifying and differentiating pulmonary dysfunction and acid–base imbalances induced by *Cp*.

**Methods:**

Forty-two calves were grouped in (i) animals inoculated with *Cp* (n = 21) and (ii) controls sham-inoculated with uninfected cell culture (n = 21). For pulmonary function testing, impulse oscillometry, capnography, and FRC (functional residual capacity) measurement were applied to spontaneously breathing animals. Variables of acid–base status were assessed in venous blood using both (i) traditional Henderson-Hasselbalch and (ii) strong ion approach.

**Results:**

Both obstructive and restrictive pulmonary disorders were induced in calves experimentally inoculated with *Cp*. Although disorders in respiratory mechanics lasted for 8–11 days, the pattern of spontaneous breathing was mainly altered in the period of acute illness (until 4 days post inoculation, dpi). Expiration was more impaired than inspiration, resulting in elevated FRC. Ventilation was characterised by a reduction in tidal volume (−25%) combined with an increased percentage of dead space volume and a significant reduction of alveolar volume by 10%. Minute ventilation increased significantly (+50%) due to a compensatory doubling of respiratory rate. Hyperventilatory hypocapnia at 2–3 dpi resulted in slightly increased blood pH at 2 dpi. However, the acid–base equilibrium was additionally influenced by metabolic components, i.e. the systemic inflammatory response, all of which were detected with help of the strong ion theory. Decreased concentrations of albumin (2–10 dpi), a negative acute-phase marker, resulted in a decrease in the sum of non-volatile weak acids (A_tot_), revealing an alkalotic effect. This was counterbalanced by acidic effects of decreased strong ion difference (SID), mediated by the interplay between hypochloraemia (alkalotic effect) and hyponatraemia (acidic effect).

**Conclusions:**

This bovine model was found to be suitable for studying pathophysiology of respiratory *Cp* infection and may help elucidating functional host-pathogen interactions in the mammalian lung.

## Background

*Chlamydiae* include important respiratory pathogens. In humans, infections with *Chlamydia* (*C*.) *psittaci* are typical examples of pulmonary zoonotic diseases, historically known as psittacosis (parrot fever) or ornithosis (transmitted from poultry). Taking into consideration that chlamydial infections are frequently present in cattle herds [1], the bovine species was also proven to be a natural host for *C. psittaci*[2-6]. Although a bovine model of experimentally induced *C. psittaci* infection was recently introduced by our group [7] we are still far from fully understanding the pathogenesis and consequences of *C. psittaci* infections.

However, addressing open questions by using a large animal model can offer greater clinical translational potential [8] and benefits both, human and veterinary medicine [9]. In this particular model, the respiratory tract as the target organ was chosen because there is still a lack of knowledge regarding the pathophysiology of pulmonary disorders induced by *C. psittaci*. With respect to the clinical outcome, respiratory chlamydial infections are known to be highly variable. Human and avian *C. psittaci* infections may range from clinically silence to acute respiratory and systemic illness. In human medicine, acute ‘atypical pneumonia’ is a well-known phenomenon in patients that acquired psittacosis due to zoonotic transmission [10-12]. Persistent infection with *C. psittaci*, however, was identified in humans with pulmonary emphysema and/or chronic obstructive pulmonary disease (COPD) as well as in horses with chronic recurrent airway obstruction [13,14] suggesting a pathogenetic link between chronic pulmonary inflammation and persistent infection with chlamydiae. Similar observations were reported for the bovine lung. While an acute outbreak of upper respiratory tract disease in calves was attributed to *Chlamydia*[3], chronic recurrent chlamydial infections in calves remained clinically inconspicuous but were associated with persistent peripheral airway obstruction and chronic pulmonary inflammation [15].

Our defined respiratory model of *C. psittaci* infection in calves offers the possibility to study cause-effect relationships under biologically relevant conditions, i.e. between a pathogen with a clear affinity to the respiratory system and a natural host. This particular study was allocated (i) to identify and to quantify acute respiratory dysfunction induced by *C. psittaci* in a mammalian lung comparable to the human lung in terms of volumes and airflows. To assess lung function parameters typically measured in human medicine, effort-independent and non-invasive pulmonary function techniques common in human pulmonology were applied to conscious and spontaneously breathing calves. (ii) In order to evaluate systemic consequences of pulmonary dysfunctions, acid–base imbalances were quantified and differentiated by assessing metabolites and electrolytes taking both the traditional Henderson-Hasselbalch approach and the new strong ion models [16,17] into account.

The results of this study provide new information regarding the pathophysiology of acute respiratory infection caused by *C. psittaci* with relevance for both veterinary and human medicine taking the ONE HEALTH concept into account.

## Methods

### Legal conformity and ethics statement

This study was carried out in strict accordance with European and National Law for the Care and Use of Animals. The protocol was approved by the Committee on the Ethics of Animal Experiments and the Protection of Animals of the State of Thuringia, Germany (Permit Number: 04-002/07). All experiments were done in a containment of biosafety level 2 under supervision of the authorised institutional Agent for Animal Protection. Bronchoscopy to inoculate the pathogen was strictly performed under general anaesthesia. During the entire study, every effort was made to minimise suffering.

### Animals

In this prospective and controlled study, 42 conventionally raised calves (Holstein-Friesian breed, male) were included. Animals originated from one farm where each individual calf was fed with maternal colostrum for at least three consecutive meals after birth. In the subsequent period, calves received mixed colostrum until they were purchased at the age of 14 to 28 days weighing between 42.2 and 71.2 kg (56.3 ± 6.8 kg; mean ± SD). The herd of origin was without any history of *Chlamydia*-associated health problems (regularly checked by the National Reference Laboratory for Psittacosis). In the institute calves were reared under standardised conditions (room climate: 18–20°C, rel. humidity: 60–65%) and in accordance with international guidelines for animal welfare. Throughout the entire study, nutrition included commercial whey-based milk replacers and coarse meal. Water and hay were supplied *ad libitum*. None of the given feed contained antibiotics.

### Study design

At the age of 42–64 days, 21 calves weighing 73.9 ± 7.4 kg were inoculated with 10^8^ inclusion forming units (ifu) of a bovine *C. psittaci* strain (DC 15) per calf, whereas another 21 calves (body weight: 69.3 ± 8.3 kg; mean ± SD) served as controls. Preparation of the challenge strain, procedure of intrabronchial inoculation using a flexible video-bronchoscope and scheme of inoculation at 8 defined localisations in the lung have been described elsewhere [7]. Controls were inoculated with uninfected Buffalo Green Monkey Kidney cell culture suspended in 6 mL stabilising medium SPGA (containing saccharose, phosphatile substances, glucose and bovine albumin; [18]) using the same methodology.

As illustrated in Table 1, pulmonary function tests (PFT) were performed in 18 *C. psittaci*-infected and in 18 sham-inoculated calves from 7 days *ante inoculation* (a.i.) up to 14 days *post inoculation* (dpi). Body weight (b.w.) was measured individually prior to each lung function test. Prior morning feeding blood samples were collected from the jugular vein starting 1 hour a.i. up to 14 dpi (Table 1). After blood sampling and PFT per day, three calves per group were sacrificed 2, 4, 7, 10, and 14 dpi. Consequently, the number of calves per group decreased continuously from n = 21 at the beginnig of the study to n = 6 at the end of the study (14 dpi).

**Table 1:** Study design

	**Animals**	**−7**	**−4**	**−1**	**+1**	+**2**	**+3**	**+4**	**+7**	**+8**	**+10**	**+11**	**+14**
		**d a.i.**	**d a.i.**	**h a.i.**	**dpi**	**dpi**	**dpi**	**dpi**	**dpi**	**dpi**	**dpi**	**dpi**	**dpi**
PFT	*C. psittaci*	n = 18	n = 18				n = 18	n = 15	n = 12	n = 9	n = 9	n = 6	n = 6
	Controls	n = 18	n = 18				n = 18	n = 15	n = 12	n = 9	n = 9	n = 6	n = 6
Blood	*C. psittaci*			n = 21	n = 21	n = 21	n = 18	n = 15	n = 12		n = 9		n = 6
	Controls			n = 21	n = 21	n = 21	n = 18	n = 15	n = 12		n = 9		n = 6

d a.i./h a.i., day/hour ante inoculation (baseline); dpi, day post inoculation; PFT, pulmonary function testing.

### Protocol of pulmonary function testing

All PFT measurements were performed in conscious calves breathing spontaneously through a tightly fitting facemask (dead space of facemask: < 100 mL), and in a room with controlled ambient conditions (18–20°C, rel. humidity: 60–65%). After an adaptation period of approximately 5 min, three non-invasive lung function techniques (all JAEGER, CareFusion) were applied consecutively to each animal per time point: (1) impulse oscillometry system to assess respiratory mechanics, (2) volumetric capnography to measure the concentration of exhaled CO_2_ against exhaled volume, and (3) re-breathing system to assess FRC (functional residual capacity). All systems were originally produced for human medicine and have been successfully applied to calves previously [19-22]. In each system, a Lilly-type pneumotachograph (mesh resistance: 36 Pa/(L/s)) was used for continuous measurement of airflow (V’).

#### Impulse oscillometry

Complex respiratory impedance, consisting of both respiratory resistance (Rrs) and respiratory reactance (Xrs), was analysed in the frequency range 3 Hz - 15 Hz as described elsewhere [15,22,23]. In addition, proximal airway resistance (Rprox) and distal airway resistance (Rdist) were calculated [15,21]. Three impulse oscillometry measurements were performed per calf and time point as described by Jaeger et al. (2007) [15]. Duration of one measurement was 60 seconds with 3 test impulses per second (sec), and 32 sampling points after each impulse with a period between two sampling points of 5 ms. Results of three measurements per animal and time point were averaged and these average values were used for further statistical analysis.

#### Volumetric capnography

**Figure 1:**
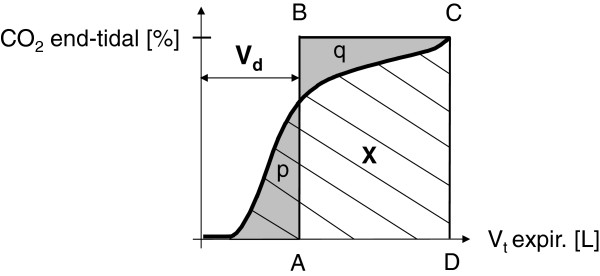
**Evaluation of dead space volume (Vd) and end-tidal CO**_**2 **_**concentration using volumetric capnography.** The rectangle **ABCD** represents the same area as the CO_2_-volume-area under the capnographic curve (X) while **BC** represents a constant CO_2_ concentration that equals to end-tidal CO_2_. In this case, areas p and q are of the same size, and Vd according to Bohr represents the volume without CO_2_.

Volumetric capnography is the projection of expired CO_2_*versus* expired volume. In a breath-by-breath analysis, 10 exhaled CO_2_ curves were registered per calf and time point in triplicate. Dead space volume and end-tidal CO_2_ were calculated for each breath as shown in Figure 1. Results of all 30 exhaled CO_2_ concentration curves per individual measurement were averaged for further statistical analyses.

#### Re-breathing method

FRC of the lung was measured by the multiple breath Helium-dilution technique (wash-in), using Helium (He) as test component of the inspired gaseous mixture (inspiratory concentrations: 7–10% He, 35% oxygen, rest nitrogen). The volume of the reservoir (re-breathing) bag was filled with 9 L of the test gas, expired CO_2_ was absorbed and O_2_ was added when the bag volume decreased. Re-breathing time to perform the test was 2 minutes ± 38 sec (mean ± SD).

All PFT parameters were calculated automatically using the software included in the three systems. For further analysis, the following variables of pulmonary function were taken into account: - complex respiratory impedance in the frequency range 3 Hz – 15 Hz; expressed as respiratory resistance (Rrs) and respiratory reactance (Xrs); each separated for inspiration and expiration, - proximal and distal airway resistance (Rprox, Rdist), - respiratory rate (RR), - time of inspiration and time of expiration (Tin, Tex), - tidal volume (Vt), - volume of minute ventilation (Vmin = Vt_*_ RR), - tidal volume in relation to body weight (Vt/kg), - airflow (V’) during in- and expiration (V’in, V’ex), - ratio between dead space volume and tidal volume (Vd/Vt), - functional residual capacity of the lung (FRC).

### Protocol of acid–base assessment

Jugular venous blood was collected (i) anaerobically in 2 mL polypropylene syringes with lyophilised electrolyte-balanced heparin (PICO 50, Radiometer Copenhagen) for immediate analysis and (ii) in 9.0 mL syringes (S-Monovette, Sarstedt AG & Co) for serum production.

#### Analysis of jugular venous blood

Heparinised blood samples were transported to the laboratory at room temperature and analysed within 10 min of collection using a combined blood-gas- and electrolyte-analyser (ABL 725, Radiometer), working with manufacturer’s standard electrodes. The following factors were measured in the venous (v) blood: pH(v), partial pressure of CO_2_ (pCO_2_(v)), and the plasma concentrations of sodium (cNa^+^), potassium (cK^+^), calcium (cCa^2+^) and chloride (cCl^-^) by ion-selective potentiometry. Plasma concentrations of glucose (cGlucose) and L-lactate (cL-lactate) were measured in the same equipment using enzymatic electrodes.

#### Serum biochemical analysis

Serum was harvested by centrifugation (3120 g for 15 min at 15°C) and stored at −20°C until analysed. Serum concentrations of total protein (biuret method) and inorganic phosphate (ammonium-molybdate) were measured spectrophotometrically (Cobas 6000, Roche/Hitachi). Capillary electrophoresis was performed to determine concentrations of albumin and globulin, as well as the globulin spectra (‘Capillarys2’, Sebia).

#### Calculated acid–base variables

The following variables were calculated using proprietary equations included in the software of the blood-gas- and electrolyte-analyser: blood pH and pCO_2_ (v), each corrected for the actual body temperature (BT) of the animal as measured rectally via digital thermometer before each blood collection (pH _BT_, pCO_2_(v) _BT_) and traditional variables of acid–base balance, i.e. bicarbonate (cHCO_3_^-^), standard bicarbonate (cHCO_3_^-^(st)), actual base excess (cBase), and standard base excess (cBase (Ecf)). The Henderson-Hasselbalch approach quantifies the unmeasured anion concentration by calculating the anion gap (AG) as follows [24-26]:

(1) $$\mathrm{AG}=\left(\mathrm{cN}a^{+}+cK^{+}\right)–\left(\mathrm{cC}l^{-}+\mathrm{cHC}{O_{3}}^{-}\right)$$

The strong ion model [16] simplified by Constable [17] provides a novel insight into the pathophysiology of mixed acid–base disorders. This approach is based on the assumption that plasma pH is a dependent variable and as such its value is determined by three independent factors: pCO_2_, the strong ion difference (SID), and the sum of non-volatile weak acids (A_tot_) [27]. SID is the difference between the total sum of all strong cation concentrations and the sum of all strong anion concentrations. Strong ions are those that dissociate completely at physiologic pH, existing either as strong cations (Na^+^, K^+^, Ca^2+^, Mg^2+^) or strong anions (principally Cl^-^ and L-lactate). The most important measured strong ions are Na^+^, K^+^ and Cl^-^, calculated in SIDm_3_[28,29].

(2) $$\mathrm{SID}m_{3}\left[\mathrm{mmol}/L\right]=\mathrm{cN}a^{+}+cK^{+}-\mathrm{cC}l^{–a}$$

All other electrolytes (measurable or unmeasurable) are involved in the strong ion gap (SIG), the difference between remaining unmeasured cations (cUC = Ca^2+^, Mg^2+^, and in very low amounts - and therefore negligible - micronutrients e.g. Cu ^2+^, Fe^2+^, Fe^3+^, Zn^2+^, Co^2+^, Mn^2+^) and unmeasured anions (cUA = SO_4_^2-^, lactate^-^, beta-hydroxybutyric acid^-^, acetoacetic acid^-^) [30].

(3) SIG=cUC−cUA

Re-arranging gives:

(4) $$\mathrm{SID}=\mathrm{SID}m_{3}+\mathrm{SIG}\;\mathrm{or}$$

(5) $$\mathrm{SID}=\left(\mathrm{cN}a^{+}+cK^{+}+\mathrm{cUC}\right)–\left(\mathrm{cC}l^{-}+\mathrm{cUA}\right)$$

The SID represents the net charge which must be balanced by charges on the weak acids in the solution for electrical neutrality to be maintained [17]. Acid total (A_tot_) represents the total amount of non-volatile weak acid present in the system. The law of conservation of mass means that the total amount of A_tot_ in the system must be constant [31]. In plasma, the major non-volatile weak acids present are plasma proteins and phosphates [32,33]. In calves, however, the albumin concentration (cAlbumin) is most important and can be used alone as an estimate of A_tot_ in plasma, the results of which were used to calculate and prepare a gamblegram [34]. A_tot_ and SIG were calculated from both total protein concentration (cProtein total) and cAlbumin and temperature corrected blood pH using the following equations and pK_a_ data for calves [25,26,28]:

(6) $$A_{\mathrm{tot}\;\left(\mathrm{Alb}\right)}\left[\mathrm{mmol}/L\right]=0.622\times \mathrm{cAlbumin}\;\left[g/L\right]$$

(7) $$A_{\mathrm{tot}\;\left(\mathrm{Prt}\right)}\left[\mathrm{mmol}/L\right]=0.343\times \mathrm{cProtein}\;\mathrm{total}\;\left[g/L\right]$$

(8) $$\mathrm{SIG}=A_{\mathrm{tot}}/\left(1+10^{\left(\mathrm{pKa}-\mathrm{pH}\right)}\right)–\mathrm{AG}$$

with pK_a_ = 7.08 [K_a_ = (0.84 ± 0.41) × 10^-7^].

(9) $$\begin{array}{ll} \mathrm{SI}G_{\left(\mathrm{Alb}\right)}\left[\mathrm{mmol}/L\right]= & \mathrm{cAlbumin}\;\left[g/L\right]\\ & \times (0,622/\left(1+10^{\left(7,08-\mathrm{pH}\right)}\right)-\mathrm{AG} \end{array}$$

(10) $$\begin{array}{ll} \mathrm{SI}G_{\left(\mathrm{Prt}\right)}\left[\mathrm{mmol}/L\right]= & \mathrm{cProtein}\;\mathrm{total}\;\left[g/L\right]\\ & \times (0,343/\left(1+10^{\left(7,08-\mathrm{pH}\right)}\right)-\mathrm{AG} \end{array}$$

### Statistical methods

Normally distributed data are presented as mean and standard deviation (SD) while data with unknown or non-normal distribution are given as median and range. The analysis of lung function data was performed using PASW (Predictive Analyse Software) Statistics 17.0 (IBM Corporation) and StatgraphicsPlus 4.0 (StatPoint Technologies, Inc.). To compare multiple data with normal distribution, multifactorial analysis of variance (ANOVA) was used with Bonferroni's multiple comparison procedure as *post hoc* test. To compare two unpaired samples, i.e. differences between two groups at one time point, the unpaired *t*-test was used for normally distributed data (comparison of means) while the Mann-Whitney-Wilcoxon *W* test was used for data with unknown or non-normal distribution (comparison of medians).

For analysis of acid–base variables, Matlab (Matlab R2007a, Version 7.4.0.287; The MathWorks, Inc.) was used. Significant changes within each group compared to baseline data were assessed by Wilcoxon signed rank test, while Mann-Whitney-Wilcoxon *W* test was used to identify significant differences between groups at a given time point [35,36]. Since the given p are equal or less than 0.05, there is a statistically significant difference at the 95.0% confidence level. All confidence levels (p) are given with the results.

## Results

### Respiratory mechanics

**Figure 2:**
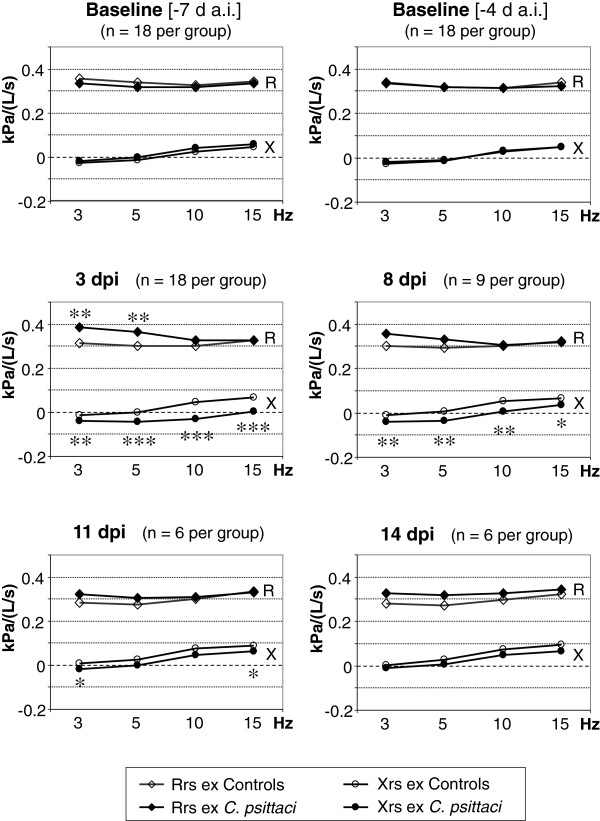
**Respiratory impedance during expiration.** Complex respiratory impedance during expiration is expressed as resistance (R) and reactance (X). d a.i., days after inoculation; dpi, days post inoculation; Rrs ex, respiratory resistance during expiration; Xrs ex, respiratory reactance during expiration. Open symbols: control calves. Filled symbols: calves inoculated with *C. psittaci*. Data are expressed as means per group. Significant differences between groups are indicated by *(p < 0.05), **(p < 0.01), or ***(p < 0.001) [unpaired *t*-test]. For reasons of visual clarity, data are expressed as means per group only. Numeric data presenting standard deviation and results of within-group comparison in addition are given in Table 2.

Before challenge, complex respiratory impedance assessed by impulse oscillometry was comparable between groups and reproducible within each group (Figure 2; baseline data). After inoculation of *C. psittaci*, Xrs - representing the elastic properties of the lung - decreased significantly at all frequencies (3–15 Hz) compared to control calves. Coevally, respiratory resistance at low frequencies (Rrs ≤ 5 Hz) increased significantly. This effect was stronger during expiration (Figure 2) than inspiration (data not shown). Figure 2 illustrates frequency-dependent courses of respiratory impedance assessed during expiration at selected time points indicating that significant differences in Xrs between groups lasted for at least 11 dpi. Numeric data (given in Table 2 for selected time points corresponding to Figure 2) and within-group analysis over time revealed a continuous growth-related increase in Xrs within the observation period of 21 days in controls that was clearly absent in calves exposed to *C. psittaci*. Instead, Xrs decreased significantly 3 dpi compared to intra-group baseline data before inoculation of *C. psittaci* (Table 2).

**Table 2:** **Respiratory impedance assessed during expiration in the frequency range 3–15 Hz in calves either challenged with 10**^**8 **^**inclusion forming units of *C. psittaci *** **or sham-inoculated controls**

		**−7 d a.i.**	**−4 d a.i.**	**3 dpi**	**8 dpi**	**11 dpi**	**14 dpi**
		n = 18 per group	n = 18 per group	n = 18 per group	n = 9 per group	n = 6 per group	n = 6 per group
**Xrs ex 3 Hz**	*C. psittaci*	−0.018	−0.021	**−0.038**	**−0.040**	**−0.018**	−0.010
		[0.015]	[0.012]	[0.031]	[0.026]	[0.019]	[0.017]
	Controls	−0.026	−0.028	−0.014	- 0.009	0.006	0.004
		[0.026]	[0.028]	[0.017]	[0.019]	[0.015]	[0.011]
**Xrs ex 5 Hz**	*C. psittaci*	−0.003	−0.009	**−0.045** ↓	**−0.034**	0.000	0.007
		[0.018]	[0.015]	[0.041]	[0.032]	[0.023]	[0.022]
	Controls	−0.015	−0.015	0.001	−0.008	0.025	0.026
		[0.035]	[0.032]	[0.021]	[0.025]	[0.022]	[0.020]
**Xrs ex 10 Hz**	*C. psittaci*	0.041	0.027	**−0.031** ↓	**0.005**	0.045	0.047
		[0.024]	[0.025]	[0.050]	[0.032]	[0.023]	[0.041]
	Controls	0.023	0.032	0.046	0.051	0.077	0.075
		[0.045]	[0.038]	[0.031]	[0.033]	[0.033]	[0.032]
**Xrs ex 15 Hz**	*C. psittaci*	0.058	0.049	**0.002** ↓	**0.037**	**0.064**	0.066
		[0.028]	[0.026]	[0.046]	[0.023]	[0.018]	[0.035]
	Controls	0.047	0.050	0.068	0.067	0.089	0.093
		[0.034]	[0.036]	[0.032]	[0.030]	[0.017]	[0.021]
**Rrs ex 3 Hz**	*C. psittaci*	0.333	0.334	**0.387**	0.355	0.321	0.329
		[0.051]	[0.038]	[0.103]	[0.059]	[0.052]	[0.074]
	Controls	0.335	0.339	0.314	0.303	0.284	0.279
		[0.068]	[0.060]	[0.044]	[0.059]	[0.038]	[0.031]
**Rrs ex 5 Hz**	*C. psittaci*	0.318	0.319	**0.366**	0.329	0.306	0.319
		[0.047]	[0.036]	[0.096]	[0.046]	[0.048]	[0.071]
	Controls	0.337	0.318	0.300	0.291	0.276	0.272
		[0.060]	[0.050]	[0.040]	[0.056]	[0.031]	[0.033]
**Rrs ex 10 Hz**	*C. psittaci*	0.319	0.314	0.326	0.306	0.310	0.327
		[0.042]	[0.031]	[0.079]	[0.038]	[0.035]	[0.061]
	Controls	0.328	0.314	0.302	0.302	0.299	0.296
		[0.044]	[0.038]	[0.034]	[0.047]	[0.014]	[0.038]
**Rrs ex 15 Hz**	*C. psittaci*	0.336	0.321	0.325	0.319	0.330	0.343
		[0.041]	[0.027]	[0.074]	[0.043]	[0.040]	[0.055]
	Controls	0.344	0.339	0.327	0.324	0.335	0.324
		[0.041]	[0.040]	[0.032]	[0.035]	[0.020]	[0.035]

d a.i., days *ante inoculation*; dpi, days post inoculation; Rrs ex, respiratory resistance during expiration; Xrs ex, respiratory reactance during expiration. Data are given as mean [standard deviation]. Significant difference between groups at the given time point are highlighted in bold (unpaired *t*-test, p ≤ 0.05). ↓ indicates a significant decrease compared to baseline data within one group (ANOVA, *post hoc* test: Bonferroni's multiple comparison procedure, p ≤ 0.01).

**Figure 3:**
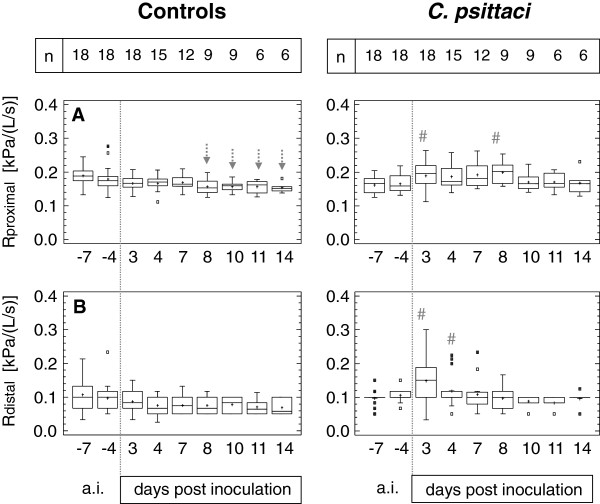
**Proximal and distal airway resistance.** Calves were either challenged with 10^8^ inclusion forming units of *C. psittaci* or sham inoculated (controls). Rdistal, distal airway resistance; Rproximal, proximal airway resistance. Data are expressed as Box-and-Whisker Plots representing lower and upper quartile values (box) with median and mean (+). Whiskers extend from each end of the box to the most extreme values within 1.5 interquartile ranges. Outliers are data beyond the ends of the whiskers. In calves inoculated with *C. psittaci*, # indicates a significant difference at the given time point compared to controls (Mann-Whitney-Wilcoxon *W* test) at a probability level of p ≤ 0.05. In controls, arrows indicate significant decreases (↓) at the given time point compared to data assessed within the group −7 days *ante inoculationem* (a.i.) at a probability level of p ≤ 0.05 (ANOVA, *post hoc* test: Bonferroni's multiple comparison procedure).

Proximal and distal airway resistances (Rprox, Rdist) are given in Figure 3A-B. Calves exposed to *C. psittaci* showed significantly elevated Rprox and Rdist data compared to controls as well as compared to intra-individual baseline data. Interestingly, the increase in Rdist was larger (146%; mean of intra-subject difference between baseline a.i. and 3 dpi) compared to the increase in Rprox (116%), but duration of significantly-elevated airway resistance lasted longer in proximal airways (until 8 dpi) compared to distal airways (4 dpi).

### Respiratory pattern

**Figure 4:**
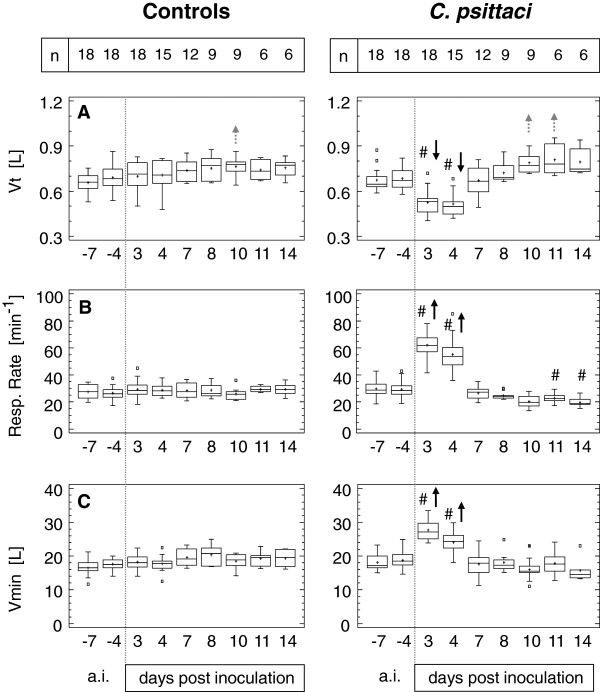
**Variables of the spontaneous breathing pattern.** Calves were either challenged with 10^8^ inclusion forming units of *C. psittaci* or sham inoculated (controls). RR, respiratory rate; Vmin, volume of minute ventilation; Vt, tidal volume. Data are expressed as Box-and-Whisker Plots representing lower and upper quartile values (box) with median and mean (+). Whiskers extend from each end of the box to the most extreme values within 1.5 interquartile ranges. Outliers are data beyond the ends of the whiskers. In calves inoculated with *C. psittaci*, # indicates a significant difference at the given time point compared to controls (Mann-Whitney-Wilcoxon *W* test) at a probability level of p ≤ 0.01. Arrows (↑ or ↓) indicate significant increases or decreases, respectively, at the given time point compared to data *ante inoculationem* (a.i.) within the group (ANOVA, *post hoc* test: Bonferroni's multiple comparison procedure) at a probability level of p ≤ 0.05 (grey) or p ≤ 0.01 (black).

The pattern of spontaneous breathing was characterised by the variables given in Figure 4. In controls, tidal volume (Vt) increased continuously due to growth over time. Averaged respiratory rate (RR) was 28 breathing cycles per minute and did not change during the study. Thus, minute ventilation (Vmin) increased slightly (but not significantly) from 16.5 L (mean −7 days) to 19.4 L (mean 14 dpi). In calves exposed to *C. psittaci*, significant alterations in the respiratory pattern were seen 3–4 dpi compared to baseline data, characterised by a reduction of Vt by 25% and a doubling of RR. Consequently, Vmin increased to about 150% of baseline. These changes were reversed by 10 dpi and, compared to intra-group data before challenge, Vt was significantly increased while RR was significantly decreased.

**Figure 5:**
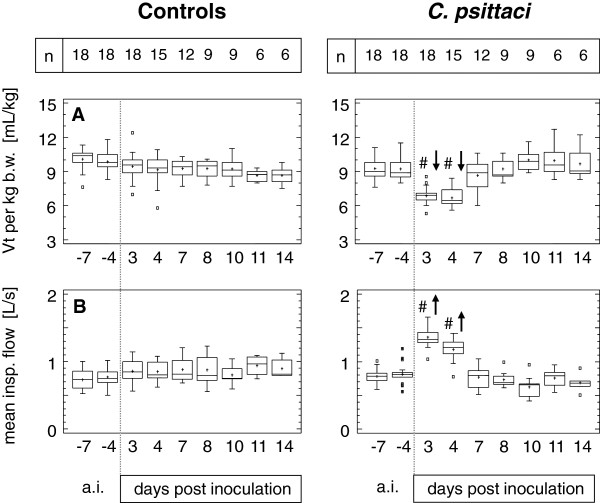
**Tidal volume per kg body weight and mean inspiratory airflow.** Calves were either challenged with 10^8^ inclusion forming units of *C. psittaci* or sham inoculated (controls). b.w, body weight; Vt, tidal volume. Data are expressed as Box-and-Whisker Plots representing lower and upper quartile values (box) with median and mean (+). Whiskers extend from each end of the box to the most extreme values within 1.5 interquartile ranges. Outliers are data beyond the ends of the whiskers. In calves inoculated with *C. psittaci*, # indicates a significant difference at the given time point compared to controls (Mann-Whitney-Wilcoxon *W* test) at a probability level of p ≤ 0.01. Arrows (↑ or ↓) indicate significant increases or decreases, respectively, at the given time point compared to data *ante inoculationem* (a.i.) within the group (ANOVA, *post hoc* test: Bonferroni's multiple comparison procedure) at a probability level of p ≤ 0.01.

To eliminate any influence of growth from volumes of respiration, Vt was additionally calculated per kg b.w. (Figure 5A). In calves challenged with chlamydiae, minima of 6.9 and 6.7 mL/kg (means) were measured at 3 and 4 dpi, while averaged Vt per kg b.w. ranged between 8.7 mL/kg and 10.1 mL/kg in controls and in challenged calves at other time points.

With doubling of RR in *C. psittaci*-exposed calves, time of inspiration (Tin) and time of expiration (Tex) were significantly shorter compared to baseline data before challenge (Tin: 0.44 sec 3 dpi compared to 1.0 sec at baseline; Tex: 0.56 sec 3 dpi compared to 1.2 sec at baseline; means). The mean ratio Tex:Tin, however, ranged within each group between 1.2 and 1.3 without changing significantly in any group at any time point (data not shown in detail).

Airflows assessed during inspiration and expiration (V’in, V’ex) revealed that mean inspiratory flow was physiologically higher (0.8 L/s) compared to expiratory flow (0.6 L/s) at baseline in both groups. Due to inoculation of chlamydiae, increases in airflows at time points 3 dpi and 4 dpi were comparable during in- and expiration (about 170% at 3 dpi and 140% at 4 dpi compared to baseline data). Data are only depicted for V’in (Figure 5B).

### Alveolar ventilation

**Figure 6:**
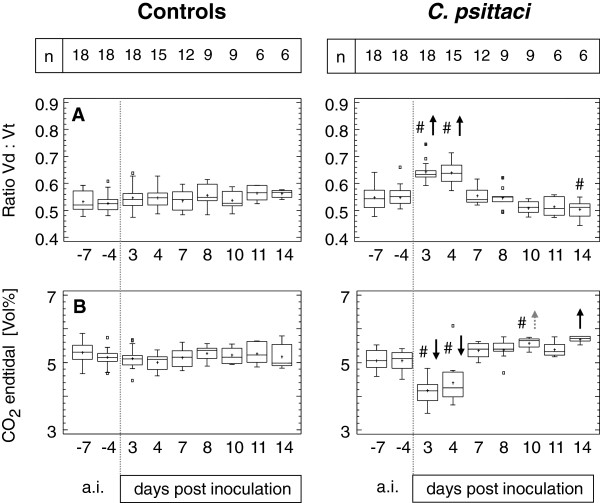
**Dead space volume in relation to tidal volume and end tidal concentration of CO**_**2**_**.** Calves were either challenged with 10^8^ inclusion forming units of *C. psittaci* or sham inoculated (controls). Vd, dead space volume; Vt, tidal volume. Data are expressed as Box-and-Whisker Plots representing lower and upper quartile values (box) with median and mean (+). Whiskers extend from each end of the box to the most extreme values within 1.5 interquartile ranges. Outliers are data beyond the ends of the whiskers. In calves inoculated with *C. psittaci*, # indicates a significant difference at the given time point compared to controls (Mann-Whitney-Wilcoxon *W* test) at a probability level of p < 0.01. Arrows (↑ or ↓) indicate significant increases or decreases, respectively, at the given time point compared to data *ante inoculationem* (a.i.) within the group (ANOVA, *post hoc* test: Bonferroni's multiple comparison procedure) at a probability level of p ≤ 0.05 (grey) or p ≤ 0.01 (black).

The mean ratio between dead space volume and tidal volume per breath (Vd:Vt, Figure 6A) was about 0.54 in controls as well as in calves before challenge with chlamydiae. Three and 4 dpi of *C. psittaci*, the percentage of Vd per breath increased to 64% (Vd:Vt = 0.64) in average indicating that alveolar volume per breath was reduced by about 10%. FRC (i.e. the volume present in the lung at end of spontaneous expiration) was significantly increased by about 500 mL in the *C. psittaci* group. While FRC was 3.0 L (40.5 mL/kg b.w.) at baseline, it was elevated to 3.5 L (45.7 mL/kg b.w.) 3 dpi after inoculation of chlamydiae (ANOVA, LSD, p < 0.01). In control animals neither a significant increase of FRC over time was seen nor a decrease of end-tidal CO_2_. End-tidal (i.e. alveolar) concentration of CO_2_ in exhaled breath decreased significantly from about 5 Vol% (baseline data in both groups) to 4.2 or 4.4 Vol%, respectively, at 3–4 dpi in calves exposed to *C. psittaci* (Figure 6B).

### Blood gases and acid–base variables

During the acute phase of infection and the development of respiratory illness, pCO_2_ in venous blood (pCO_2_(v) _BT_) of calves inoculated with *C. psittaci* was significantly reduced 2–3 dpi while blood pH was significantly increased 2 dpi compared to baseline values within the challenge group and compared to non-infected control calves at the same time points (Additional file 1). In the same time period, both cHCO_3_^-^ (st) and cBase (Ecf)) were significantly elevated 2 dpi while all bicarbonate and base excess data (cHCO_3_^-^, cHCO_3_^-^(st), cBase, cBase (Ecf)) were significantly reduced one day later (3 dpi).

After a significant rise of SIDm_3_ at 1 dpi, all strong ion differences (SIDm_3_, SIDm_4_, SIDm_5_) also dropped significantly down at 3 dpi compared to baseline data as well as compared to SIDm_3_, SIDm_4_, SIDm_5_ data assessed in controls (Additional file 2). Calculated anion gap (AG) was significantly elevated within the first 3 days after *C. psittaci* challenge while strong ion gap (SIG) was significantly reduced between 1–4 dpi (Additional file 1). The latter effect was more prominent in SIG _(Alb)_ compared to SIG _(Prt)_ due to significantly diminished A_tot (Alb)_ values between 2–10 dpi compared to baseline data before challenge and compared to non-infected controls (Additional file 3). During the resolution period of clinical signs (about one week after inoculation), pCO_2_(v) _BT_ was significantly increased in *C. psittaci* infected calves compared to baseline data before challenge (7 dpi, 10 dpi) and compared to data obtained from non-infected calves (7 dpi) without any marked changes in blood pH. Within the period 7–10 dpi, cHCO_3_^-^, cHCO_3_^-^(st), cBase, and cBase (Ecf) were significantly increased compared to baseline data and compared to data obtained from control calves (Additional file 1). SIDm_3_, SIDm_4_ and SIDm_5_ started to increase significantly within the infected group at 7 dpi, and were even higher 10–14 dpi (significant in comparison to both baseline values and control calves) (Additional file 2). While the significant reduction in A_tot (Alb)_ lasted until 10 dpi, A_tot (Prt)_ was slightly increased 10–14 dpi in calves exposed to the pathogen (Additional file 3). Thus, in comparison to non-infected calves, SIG _(Prt)_ of calves exposed to *C. psittaci* was higher at 7 dpi while SIG _(Alb)_ was lower at 10 dpi (Additional file 1).

### Serum biochemical analysis and protein electrophoresis

Blood concentration of inorganic phosphate of calves infected with *C. psittaci* decreased significantly between 1–10 dpi (compared to baseline data) with minima between 2–7 dpi that were also significantly lower than in controls (Additional file 3).

Serum albumin concentration was significantly reduced from 2 dpi till 10 dpi in infected calves (compared to baseline values and compared to control calves) with a maximal reduction observed at 4–7 dpi. In contrast, concentration of serum globulins increased significantly over time after experimentally induced infection (different time courses of single globulin fractions as given in Additional file 3). Consequently, the concentration of serum proteins measured in calves infected with *C. psittaci* was, compared to baseline data, significantly decreased at 2–3 dpi and significantly increased at 10–14 dpi. The ratio between albumin and globulin in the infected group, however, was significantly decreasing in the course of the study (Additional file 3).

### cGlucose, cL-lactate and electrolytes

Concentration of blood glucose was significantly reduced during the first 7 days after *C. psittaci* challenge with a lowest group median seen at 3 dpi. In contrast, cL-lactate in venous blood was significantly increased within the period 1–4 dpi with a highest group median seen at 2 dpi. Changes of cGlucose and cL-lactate in *C. psittaci*-inoculated calves were significant in comparison to both baseline data and control calves (Additional file 2). With respect to electrolytes in the peripheral blood, the concentrations of calcium, sodium, and chloride were slightly, but significantly, reduced in inoculated calves after challenge (cCa^2+^: 2–4 dpi, cNa^+^: 2–7 dpi, cCl^-^: 2–14 dpi). Potassium concentration (cK^+^) was, compared to baseline values and compared to control calves, significantly increased at 1 dpi and significantly decreased at 4 dpi after inoculation of *C. psittaci* (Additional file 2).

### Strong ion approach of acid–base disorders

**Figure 7:**
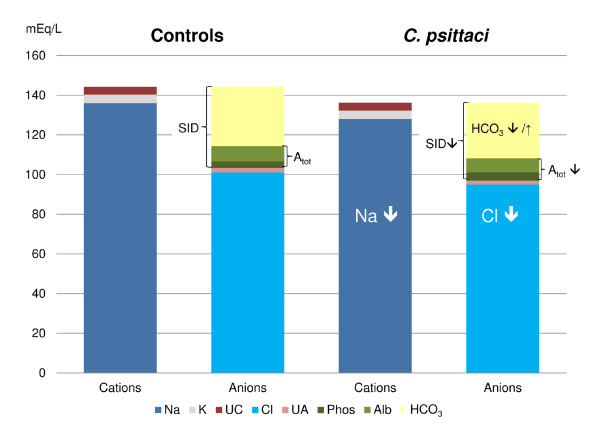
**Gamblegram at 3 dpi.** Calves were either challenged with 10^8^ inclusion forming units of *C. psittaci* or sham inoculated (controls). The thickness of the arrow indicates the strengths of the influence. Na^+^ decreased remarkably (8 mmol/L) 3 dpi in *C. psittaci* infected calves, therefore HCO_3_^–^ decreased; but Cl^-^ decreased clearly (6 mmol/L) too, therefore HCO_3_^–^ should increase. Both influences together Na^+^ and Cl^-^ lead to a minimal decrease of HCO_3_^–^. Similar reaction were seen in SID and A_tot_, both decreased (SID = acidotic, A_tot_ = alkalotic), that is why no change occurred in venous pH.

Figure 7 provides an example of the interplay of the afore-mentioned components during the acute phase (3 dpi) of the disease course. Decreases of cNa^+^ and cCl^-^ influenced cHCO_3_^-^ in an opposite manner. Similarly, the decrease of SID (acidotic effect) counterbalanced the decrease of A_tot_, (alkalotic effect), thus effects of these parameters on blood pH (Additional file 1) appeared minimal.

## Discussion

This prospective controlled study was undertaken to evaluate pulmonary dysfunctions induced by *C. psittaci* in the mammalian lung along with systemic acid–base alterations and imbalances in electrolytes and metabolites. A bovine model was exploited for the following reasons. (i) The lungs of species lacking collateral airways (predominantly cattle and pigs) are extremely sensitive to functional consequences of any changes in respiratory mechanics [37].

Thus, the bovine lung does present an advantageous model to assess pathophysiological consequences of both airway obstructions and pulmonary restrictions. (ii) This large animal model offers the great potential to perform non-invasively and almost painless long-term studies allowing a simultaneous within-subject approach of functional changes of both the organ and the systemic level.

In this particular model signs of acute respiratory illness were maximal 2–4 dpi and did not last longer than one week after challenge [38]. Although the severity of illness was mainly driven by respiratory signs, additional systemic reaction, similar to atypical pneumonias in human medicine [39] were observed. The most striking symptoms included dry cough, tachypnea, fever, reduced appetite, and tachycardia [38].

It was also shown earlier that in this model respiratory insufficiency during the acute phase (2–3 dpi) is characterised by hypoxaemia, linked to reduced haemoglobin oxygen saturation, increased alveolar-arterial oxygen partial pressure difference and pulmonary shunt [40]. Due to long time course of the present study the risk of secondary infection after catheterising a representative arterial vessel was not taken, as anticipated advantages with respect to the evaluation of acid–base disorders and PFTs were thought to be minimal.

### Pulmonary dysfunctions

Pulmonary function techniques from human medicine were applied to spontaneously breathing animals with body weights comparable to adult humans. Thus basic parameters of pulmonary functions (for example airflows and lung volumes) were more comparable and transferable to human patients compared to results obtained from murine models.

Furthermore, a functional differentiation between airway resistance and tissue mechanics of the lung was possible in the present study, while assessment of compliant properties of the lung in mice would require medical or surgical treatment [41,42]. During the acute period of illness (3–4 dpi) due to *C. psittaci* infection, the pattern of breathing was characterised by a significant decrease in tidal volume and a significant increase in both respiratory rate and airflows (clinically seen as short and rapid breathing cycles, i.e. dyspnoea). Per breath, dead space volume was significantly elevated while alveolar volume was reduced by about 10% indicating alveolar hypoventilation that was confirmed by a decreasing end-tidal concentration of CO_2_. However, global hypoventilation was not confirmed. In contrast, partial pressure of CO_2_ in peripheral blood decreased, too, indicating hypocapnia due to global hyperventilation. The latter was caused by an increase in minute ventilation by 50% due to the strong increase in respiratory rate. The elevated minute ventilation was most likely the attempt to compensate for hypoxaemia induced by *C. psittaci* infection as shown previously [40].

Alterations in respiratory mechanics after inoculation of *C. psittaci* included both obstructive and restrictive components and lasted longer than the clinically visible changes in the pattern of respiration. Restriction was assessed by decreasing respiratory reactance (Xrs) which indicates limitations in elasticity or compliance of the lung-thorax system [23]. This loss in elasticity was predominantly a result of inflammatory reactions, such as cell infiltration, accumulations of fibrin and protein-rich fluid or signs of regeneration described for this model in detail elsewhere [7,43]. In the present study, the statistically significant decrease of Xrs at all frequencies (3–15 Hz) in *C. psittaci* challenged calves continued until 11 dpi. Thus, the duration of reduced compliant properties of lung tissue exceeded the presence of acute clinical signs [38] by about one week.

In the acute phase of respiratory illness, the loss of pulmonary compliance was most likely accompanied by stiffness of the peripheral respiratory system due to small airways narrowing or constriction [44]. Indeed, peripheral airflow was limited in calves inoculated with *C. psittaci* compared to control calves during the acute phase of disease which was indicated by an increase of Rrs ≤ 5 Hz and Rdist (significant at 3 dpi and at 3–4 dpi, respectively). The negative frequency dependence of Rrs, i.e. an increase only at low frequencies (Rrs < 5 Hz), is a valid diagnostic tool to identify peripheral airways obstruction in both humans [45] and calves [21]. In addition to obstruction in distal airways, calves experimentally challenged with *C. psittaci* also suffered from obstruction in central or upper airways as indicated by an increase Rprox. These finding are in good agreement with reports in literature associating chlamydial infections in calves with both upper respiratory tract disease [3] and obstruction of peripheral airways [15]. Moreover, it was also shown in experimentally *C. suis* challenged pigs that peripheral airways obstruction during the acute phase (3 dpi) were followed by upper airways obstruction (at 7 dpi). To our knowledge lung function data of humans suffering from acute chlamydial pneumonia are not available, but taking these findings together obstruction of the upper and lower respiratory tract might probably also be involved in pathogenesis of acute chlamydial pneumonia in humans.

In parallel to the presence of airways obstruction, FRC increased significantly at 3–4 dpi. Baseline data of about 40 mL/kg b.w. measured in this study in calves are in good agreement with data reported for the healthy bovine lung in adult cows (38.6 ± 3.1 mL/kg; [46]). After *C. psittaci*-infection, FRC increased significantly by 17% to 45.7 mL/kg b.w. (3 dpi) which is moderate compared to FRC data reported in cows with severe bronchiolitis and an expanded lung field (56.5 ± 7.7 mL/kg; [47]). In calves, due to the lack of collateral airways, the presence of fibrin, inflammatory cells, detritus and protein rich fluid in the airways and/or alveoli during the acute phase of this model [43] resulted in narrowed peripheral airways which can easily result in the development of trapped air. In the present model the increase in FRC was transient, thus hyperinflation or over-distension of alveoli is indicated rather than the presence of emphysema [48]. As over-distension might reduce the recoil of elastic fibers it is likely that hyperinflation also contributed to reduced lung compliance described above. An increase of FRC was also reported for *C. suis* infected swine [49]. Radiographically-impressive distension of the lung with air is found in cases of *C. trachomatis* pneumonia in children, which despite the mild respiratory symptoms in infancy is associated with obstructive limitations up to 7–8 years after hospitalisation (i.e. increased FRC, forced and peak expiratory flow rates) [50,51]. A long-term impairment of lung function and structure after chlamydial infection was also shown for naturally *Chlamydia*-infected calves [15] and experimentally challenged mice [52]. In human medicine, asthma is a common chronic inflammatory disease of the airways, and the involvement of *C. pneumoniae* in asthma pathogenesis is still largely discussed [53,54].

None of the lung functions assessed in control animals was significantly influenced by intrabronchial inoculation of BGM cell suspension. Pulmonary function data in control calves revealed physiological changes over time due to lung growth and development (gain in body weight during the study was 0.6 kg per day in average). In control calves Xrs increased significantly over time, displaying increasing compliant properties of lung and thorax. These findings are in line with fundamental understanding from the very beginning of veterinary pulmonology showing that lungs are easier to stretch with enhanced body or lung size [55,56]. It has been shown for growing calves that Xrs increased with increasing body weight [57]. During the period of pulmonary maturation (until a body weight of about 300 kg [58,59]) bronchiolar diameters were also shown to increase [60], resulting in decreased airway resistance.

### Acid–base imbalances

Compared to other studies [28,61-63] the control values of pH, pCO_2_, HCO_3_^–^, base excess and A_tot (Alb)_ or A_tot (Prt)_ are in the ranges reported whereas AG and SIDm_3_,_4_ were lower and SIG _(Alb)_ or SIG _(Prt)_ were higher in absolute values than those described in literature. In calves experimentally infected with *C. psittaci*, most of the effects assessed in venous blood were slight or moderate in amplitude and were mostly related to either the acute phase (2–4 dpi) or the resolution phase (7–10 dpi) after inoculation of the pathogen. Nevertheless the investigated parameters accurately assessed the influence of *C. psittaci* on the acid–base balance of the host organism.

Partial pressure of CO_2_ provides information regarding ventilation or respiratory component of acid–base balance in the Henderson-Hasselbalch equation as well as in the strong ion approach. Despite no access to arterial blood in this study, venous blood was informative enough identifying venous hypocapnia (pCO_2_(v)↓) 2–3 dpi as a result of hyperventilation (the latter was proved by pulmonary function testing). In general, hyperventilation can be caused primary by stimulation of pulmonary nociceptive receptors related to pulmonary disease and impairment of gas exchange (hypercapnia, hypoxaemia) or secondary for recovery from metabolic acidosis [64]. Natural compensatory mechanisms probably never overcompensate, and as a general rule, the pH will vary in a direction similar to the primary component disorder [65]. Therefore, it is more plausible that hyperventilation occurred to compensate for hypoxaemia, a known consequence of experimentally induced pulmonary disease in this model as reported previously by our group [40]. As a result, blood pH increased slightly 2 dpi. Decreases in both cHCO_3_^-^ and cHCO_3_^-^(st) at 3 dpi, together with decreased cBase and cBase (Ecf), can traditionally be interpreted as compensatory mechanisms to return to normal pH. In the period 7–10 dpi, cHCO_3_^-^, cBase and cBase (Ecf) increased but pH was not influenced. In conformity with the more modern approach, cHCO_3_^-^, cBase and cBase (Ecf) are described as dependent (strong ion) variables that cannot be regulated independently of pCO_2_, while SID and A_tot_ are independent variables [66]. Only the independent variables influence the system and they are not influenced by the system. A_tot_ and SID reflect the metabolic system. A_tot (Alb)_ decreased 2–10 dpi and produced an alkalotic effect caused by hypo-albuminaemia. Albumin is a negative acute-phase protein, i.e. a marker of inflammation. This finding supplements our previously reported results identifying LBP (lipopolysaccharide binding protein) as a suitable marker of the acute phase in bovines [38,40]. In addition, albumin is the most important buffer in plasma [29]. A_tot (Prt)_ was less affected because of hyper(gamma)globulinaemia, a spontaneous immune response. Our findings are in good agreement with data reported recently by Poudel et al. (2012) demonstrating that both the lowered plasma albumin and the increased globulin concentrations were associated with the intensity of *C. pecorum* infection in calves, and were attributed to ongoing systemic inflammation and its detrimental effects on liver function caused by chlamydiae [67].

SIDm_3_, SIDm_4_ and SIDm_5_ decreased 3 dpi (acidotic effect) caused by hyponatraemia (without change in haematocrit; data not shown) which dominated the concurrent hypochloraemia (alkalotic effect). SIDm_3_, SIDm_4_ and SIDm_5_ increased slightly 10–14 dpi due to continuing hypochloraemia and normalised sodium concentrations which led to a mild alkalotic effect as seen at the same time in a slightly increased pH. Effects of increased potassium and lactate were compensated and were not seen in SIDm_3_. The adaptive retention of acid during sustained hypocapnia is normally accompanied by a loss of sodium into the urine [68]. To maintain the electroneutrality in blood in the presence of hyponatraemia, the HCO3^–^ concentration must decrease concurrently. The measured values of cNa^+^ and base excess agree with studies by Funk (2007) [34], which showed that a decrease of sodium by 10 mmol/L explains a decrease of Base excess by −3 mmol/L.

This experimental study demonstrated again that often multiple acid–base disturbances exist concurrently and that mixed acid–base disturbances traditionally cannot be detected when the blood pH is unchanged. Strong ion theory provides evidence about the presence of acid–base imbalances, but only the selective view on the single parameters, which are required to calculate the strong ion variables, help to understand the complex response of the host organism and interactions between numerous variables.

## Conclusions

The present study improved the current understanding of the pathophysiology of respiratory *C. psittaci* infections. Pulmonary dysfunctions and acid–base imbalances assessed by sensitive methods lasted clearly longer than clinically obvious signs and may thus also help elucidating functional host-pathogen interactions in the mammalian lung.

## Endnotes

^a^Further analysis are based on calculations on SIDm_3_. However, to enable a better comparability to other studies SIDm_4_ and SIDm_5_ were additionally calculated:

$$\mathrm{SID}m_{4}\left[\mathrm{mmol}/L\right]=\left(\mathrm{cN}a^{+}+cK^{+}\right)-\left(\mathrm{cC}l^{–}+\mathrm{cL}-\mathrm{lactate}\right);$$

$$\mathrm{SID}m_{5}\left[\mathrm{mmol}/L\right]=\left(\mathrm{cN}a^{+}+cK^{+}+\mathrm{cC}a^{2+}\right)-\left(\mathrm{cC}l^{–}+\mathrm{cL}-\mathrm{lactate}\right).$$

Results are given in Additional file 2.

## Abbreviations

Alb: Albumin; Atot: Acid total; AG: Anion gap; BT: Body temperature; b.w.: Body weight; Ca2+: Calcium; c: Concentration; cBase (Ecf): Standard base excess; cBase: Actual base excess; Cl-: Chloride; Cp: *Chlamydia psittaci*; dpi: Days post inoculation; FRC: Functional residual capacity; He: Helium; HCO3–: Bicarbonate; HCO3–(st): Standard bicarbonate; ifu: Inclusion forming units; K+: Potassium; Ka: Acid dissociation constant; Na+: Sodium; m: Number of strong ions measured in plasma; p: Partial pressure; PFT: Pulmonary function tests; pKa: − log _10_K_a_; Prt: Protein total; Rdist: Distal airway resistance; Rprox: Proximal airway resistance; RR: Respiratory rate; Rrs: Respiratory resistance; SD: Standard deviation; SID: Strong ion difference; SIG: Strong ion gap; Tex: Time of expiration; Tin: Time of inspiration; UA: Unmeasured anions; UC: Unmeasured cations; V: Venous; V’ex: Airflow (V’) during expiration; V’in: Airflow (V’) during inspiration; Vmin: Volume of minute ventilation; Vt: Tidal volume; Xrs: Respiratory reactance.

## Competing interests

None of the authors of this paper has a financial or personal relationship with other people or organisations that could inappropriately influence or bias the content of the paper.

## Authors’ contributions

CO carried out pulmonary function tests, drafted parts of the manuscript. SL analysed variables of acid–base status, performed statistical analysis of acid–base data, drafted parts of the manuscript. CSV participated in analysis of acid–base variables and drafted parts of the manuscript. PR conceived the study, and participated in its design and coordination, supported statistical analysis of lung function data, and revised the manuscript critically.

## Authors’ information

CO is PhD student at the Dahlem Research School, graduate school of ‘Freie Universität Berlin’. SL is doctoral candidate at the Institute of Veterinary Physiology at the ‘Freie Universität Berlin’. Both students will include parts of this manuscript in their theses. CO will focus on PFT data; SL will focus on acid–base data.

## Supplementary Material

Additional file 1 **Results of blood-gas analysis, bicarbonate concentrations, base excess, anion gap, and strong ion gap. **http://respiratory-research.com/imedia/1164744271062177/supp1.xlsx. Click here for file

Additional file 2 **Concentrations of plasma glucose, L-lactate, sodium, potassium, chloride, and calculated strong ion differences (SID). **http://respiratory-research.com/imedia/1452059447106217/supp2.xlsx. Click here for file

Additional file 3 **Concentrations of inorganic phosphate and total protein, results of electrophoresis, and calculated values for A**_**(tot)**_**. **http://respiratory-research.com/imedia/5257028221062177/supp3.xlsx. Click here for file
