# Correction: Evaluation of pulmonary dysfunctions and acid–base imbalances induced by *Chlamydia psittaci* in a bovine model of respiratory infection

**DOI:** 10.1186/2049-6958-9-42

**Published:** 2014-08-06

**Authors:** Carola Ostermann, Susanna Linde, Christiane Siegling-Vlitakis, Petra Reinhold

**Affiliations:** 1 Institute of Molecular Pathogenesis at ‘Friedrich-Loeffler-Institut’ (Federal Research Institute for Animal Health), Naumburger Str. 96a, 07743 Jena, Germany; 2 Freie Universität Berlin, Faculty of Veterinary Medicine, Oertzenweg 19b, 14163 Berlin, Germany

## Correction

Following publication of our article [1], we noticed the following mistakes:

Firstly, the numbers in the headings of the additional files have been reversed. In Additional file 2, cell A1 should read “*Additional file 2*: Concentrations of plasma glucose, L‑lactate, sodium, potassium, chloride, and calculated strong ion difference (SID)” rather than “*Additional file 3*: Concentrations of plasma glucose, L‑lactate, sodium, potassium, chloride, and calculated strong ion difference (SID)”. In Additional File 3, cell A1 should read “*Additional file 3*: Concentrations of inorganic phosphate and total protein, results of electrophoresis, and calculated values for (Atot)” rather than “*Additional file 2*: Concentrations of inorganic phosphate and total protein, results of electrophoresis, and calculated values for (Atot)”.

Secondly, in the legend of Figure 2 the term “d a.i.” is not an abbreviation of “days after inoculation” and should instead read “days *ante inoculationem*”.
